# Mapping of SIVmac T-cell epitopes in cynomolgus macaques immunized with auxo-GTU-MultiSIV DNA by the intradermal route followed by electroporation

**DOI:** 10.1186/1742-4690-9-S2-P34

**Published:** 2012-09-13

**Authors:** A Corneau, B Maciel Da Silva, S Even, J Morin, N Sylla, F Martinon, A Aarninck, A Blancher, I Stanescu, R Le Grand, N Dereuddre-Bosquet

**Affiliations:** 1 Laboratoire D'Immunogénétique Moléculaire EA 3034, Faculté de Médecine, Toulouse, France; 2 FIT Biotech , Tampere, Finland; 3 CEA, Fontenay-aux-Roses, France

## Background

Intradermal immunization with electroporation using auxo-GTU-MultiHIV DNA encoding Gag, Nef, Rev and Tat induces strong and sustained T cell responses in cynomolgus macaques. Here, we used an equivalent vaccine encoding SIVmac239 antigens (Gag, Nef, Rev, Tat, Vif, Vpx and Vpr) and we described the breath of this T cell response.

## Methods

Naïve male mauritian cynomolgus macaques were immunized with auxo-GTU-MultiSIV DNA encoding Gag, Nef, Rev and Tat (n=14) or by a combination of auxo-GTU-MultiSIV DNA and auxo-GTU-SIV-vifvprvpx (n=12) by the intradermal route followed by electroporation. MHC class I and class II haplotypes were determined by microsatellite analysis. T-cell epitopes were mapped by IFN-γ ELISPOT in PBMC by using matrix of 15-mer (overlapping by 11) SIVmac239 Gag, Nef, Rev, Tat, Vif, Vpx and Vpr peptides. Sequence and length of candidate epitopes were further optimized.

## Results

Immunization induced intense and sustained IFN-γ ELISPOT responses in peripheral blood. T-cell responses against MHC class I epitopes were evidenced: two in Gag p15 (KA10(28-37) presented by Mafa-B*011:01 and EL10(58-68) presented by a MHC-Ia molecule from haplotype M3), two in Gag p27 (HL9(146-154) presented by Mafa-B*075:01 and LA9(189-197) presented by a MHC-Ib from haplotype M1), three in Nef (AS10(4-13) presented by a MHC-Ib molecule from haplotype M3; RM9(103-111) presented by Mafa-A1*063:02; LD10(146-155) presented by a MHC-Ia from haplotype M1M2M3), one in Rev (SP10(59-68) presented by Mafa-B*075:01), one in Tat (CF9(59-67) presented by Mafa-A1*063:02), four in Vif and two in Vpx. One MHC class II epitope was identified in Gag p27 (haplotype M3). Among these, to our knowledge, AF11 is a newly described epitope in cynomolgus macaques.

## Conclusion

Auxo-GTU-MultiSIV DNA vaccination followed by electroporation induced multi-epitopic T cell responses, essentially CD8+ but also CD4+. Determination of SIV T-cell epitopes in cynomolgus macaques facilitates the monitoring of specific immune responses and pre-clinical vaccine development in this model.

